# Effects of Butadiene Sulfone as an Electrolyte Additive on the Formation of Solid Electrolyte Interphase in Lithium-Ion Batteries Based on Li_4_Ti_5_O_12_ Anode Materials

**DOI:** 10.3390/polym15081965

**Published:** 2023-04-21

**Authors:** Yu-Ruei Kung, Cheng-Yao Li, Panitat Hasin, Chia-Hung Su, Jeng-Yu Lin

**Affiliations:** 1 Department of Chemical Engineering and Biotechnology, Tatung University, Taipei 104327, Taiwan; 2 Department of Chemistry and Center of Excellence for Innovation in Chemistry (PERCH-CIC), Ministry of Higher Education, Science, Research and Innovation, Faculty of Science, Kasetsart University, Bangkok 10900, Thailand; 3 Research Center for Chinese Herbal Medicine, Ming Chi University of Technology, New Taipei City 24301, Taiwan; 4 Department of Chemical and Materials Engineering, Tunghai University, Taichung City 407224, Taiwan

**Keywords:** LTO, solid electrolyte interphase, butadiene sulfone, XPS

## Abstract

In this study, butadiene sulfone (BS) was selected as an efficient electrolyte additive to stabilize the solid electrolyte interface (SEI) film on the lithium titanium oxide (LTO) electrodes in Li-ion batteries (LIBs). It was found that the use of BS as an additive could accelerate the growth of stable SEI film on the LTO surface, leading to the improved electrochemical stability of LTO electrodes. It can be supported by the BS additive to effectively reduce the thickness of SEI film, and it significantly enhances the electron migration in the SEI film. Consequently, the LIB-based LTO anode in the electrolyte containing 0.5 wt.% BS showed a superior electrochemical performance to that in the absence of BS. This work provides a new prospect for an efficient electrolyte additive for next-generation LIBs-based LTO anodes, especially when discharged to low voltage.

## 1. Introduction

Currently, the rechargeable lithium-ion batteries (LIBs) are considered as one of the most important electrochemical energy storage technologies, with high energy density, no memory effect, long lifespan, fast kinetics, and low charge loss during idle conditions, all of which can help build a greener future. Currently, LIBs have been widely used in portable devices, electric vehicles (electric vehicle; EV, hybrid electric vehicle; HEV, and plug-in hybrid electric vehicle; PHEV), and renewable energy storage due to their high energy density, outstanding cycle performance, significant safety, good reliability, and excellent rate capability. Unfortunately, there are still some issues that should be addressed, such as overcharge or discharge, temperature dependency, and the high cost of the fabrication of electrode materials [1]. Graphite is one of the anode materials in the commercial LIB. However, there are disadvantages of graphite due to the formation of dendrite and cracking at the surface of the graphite electrode when operated at high current density and low working potential (0.2 V vs. Li^+^/Li), leading to low capacity. The low working potential can cause lithium deposition, thus enhancing the formation of solid electrolyte interphase (SEI) film on its surface. The formation of SEI film is considered an important factor in an LIB, which would be associated with the cycle life, safety, and irreversible capacity of an electrode [2,3,4,5,6,7,8]. Therefore, various spinels have recently been investigated for use as the LIB anodes to replace the conventional graphite anode because of their advantage of reducing the threat of dendrite formation compared to the graphite electrode. Among them, the most popular spinel-based anode material is Li_4_Ti_5_O_12_ (LTO) because it shows a theoretical capacity of 175 mAh g^−1^, excellent Li-ion intercalation/extraction reversibility, zero strain characteristics during charge/discharge, and negligible volume change. Moreover, it also exhibits a high and flat discharge platform at 1.55 V (vs Li^+^/Li), which is higher than the reduction potential of most organic electrolytes, thus avoiding SEI film formation on the surface of LTO particles as well as ensuring a long cycling life and significant safety of the batteries. Since LTO possesses some disadvantages, such as lower electron conductivity (10^−13^ S cm^−1^) and a poor intrinsic lithium-ion diffusion coefficient (10^−8^ cm^2^ S^−1^) [9,10,11], it is considered to be inferior in anode performance due to decay in the high-rate performances. Furthermore, the SEI formation on the LTO surface was also found when it was discharged to 0.01 V. In addition, when the extensive lithium ion intercalation of LTO was found, the irreversible plat at the discharge curve around ~0.65 V was observed [12,13,14]. This leads to the irreversible capacity observed in the first and second discharge curves. Consequently, its structural instability and SEI formation at low potential restrict its application within a high cut-off voltage above 1 V, which significantly sacrifices the cell voltage and energy density. Zahib et al. [15] mentioned that spinel LTO electrode materials do not form the SEI film during the charge and discharge studies. Since the LTO electrode is generally operated at a potential more than 1.0 V, the LTO electrode is not subjected to the formation of the passivation film over the LTO electrode materials, or some other undesirable electrolyte reactions. In addition, the lack of strain in the LTO electrode materials can also improve the shelf life of the LIBs. Ahn et al. [16] reported that a few atomic layers of Al_2_O_3_ deposited on the LTO electrode improved its cycling performance (no capacity degradation after 100 cycles) and provided a higher Coulombic efficiency, compared to the standard uncoated LTO electrode, when they are cycled at low potential of 1 mV. In order to maintain the structural stability of LTO and suppress some undesirable chemical reactions, coating the surface of LTO with ultrathin oxide layers serving as a passivation film has been proposed. Shu et al. stated that the LTO is an SEI film-free anode material when it is cycled in the potential range of 1.0 and 3.0 V. Local polarization arising from uneven electrodes will result in local overcharge of 1.0 V or over-discharge of 3.0 V during the lithium uptake/release process, leading to poor performance. Since LTO is another type of coating layer on cathode materials when cycled in the potential range of 3.0–4.5 V, it is essential to study the electrode interface of the LTO/electrolyte in a broad electrochemical potential range of 0.0–5.0 V [14]. Wang et al. [17] discussed that an SEI layer on the surface of the LTO electrode has been the subject of controversy for a long time due to the delicate nature of this SEI layer and the lack of reliable characterization tools. They reported the direct visualization of SEI layer formation on an LTO electrode surface by in situ atomic force microscopy (AFM) under potential control, and the results showed that no SEI layer formed from EC/DMC based electrolyte in the potential range of 2.5–1.0 V. Extending the reduction potential down to 0 V can, however, cause the growth of an SEI layer on the LTO surface. Thus, forming an SEI layer by discharging an LTO anode down to 0 V in the first cycle, and then operating the battery in the normal potential range of 2.5–1.0 V might be a facile strategy to improve LTO battery performance. 

In recent years, many researchers have tried to improve SEI film properties by using metal oxide materials, such as Al_2_O_3_ [18,19,20], ZrO_2_ [21,22,23], and AlF_3_, deposited on the electrode surface, or by using an electrolyte additive, such as vinylene carbonate (VC) [24,25], butyl sultone [26,27], and propane sultone (PS) [28,29], dissolved in the electrolyte system. In most cases, vinyl- and oxy-group such as VC can enhance electrochemical performances such as the cycle stability and capacity utilization of LIBs [30]. For instance, Ota et al. demonstrated that VC can form a passive protective film over active electrode materials [31]. Chen et al. reported that the addition of VC as an additive can facilitate growth and stabilize the SEI film, therefore leading to the reduction of electro resistance of the SEI film [32]. Xu et al. used hetero group containing butyl sultone (C_4_H_8_SO_3_) as an additive to improve the SEI film stability, which indicated that the sulfur group could facilitate the SEI film formation with high stability, as well as significantly enhance discharge capacity and cyclic stability [27,28]. Recently, Wang et al. revealed that SEI film originated from propargyl methanesulfonate, which possesses the triple-bonded structure and the S=O functional group which can effectively form the dense SEI film, with decreased impedance. Moreover, the S=O moiety acting as a weak base site efficiently retards the reactivity of PF_5_ and reduces the decomposition of electrolytes, preventing the effect of the generation of HF and LiF upon cycling performances [33]. Moreover, the generation of a stable SEI film obtained from *p*-Toluenesulfonyl isocyanate (PTSI) on the surface of the anode materials has also been reported. The SEI film can apparently hinder further decomposition of the electrolyte, electrode erosion, and LiF formation upon cycling [34]. Moreover, the addition of electron-rich PTSI can efficiently reduce the acidic environment and suppress the reactivity of the PF_5_. Consequently, the production of HF and LiF is suppressed. Some studies also explored the effect of sulfur-containing additives on LTO or other anode materials on the cycling performance. Ugata et al. have shown that the sulfone-based electrolyte can provide slightly better discharge capacities than the conventional electrolyte in eutectic electrolytes composed of LiN(SO_2_F)_2_ and sulfones [35]. Jia et al. reported that sulfone-bearing localized high concentration electrolytes (LHCEs) show excellent compatibility with the high energy density of LIBs when combined with a proper additive. The unique solvation structure of LHCEs facilitates the synergetic decomposition of anion, solvent, and additive in the SEI formation, leading to long-term cycling performance of discharge capacity and discharge rate capability [36]. Most recently, we used propargyl methacrylate (PMA) as an efficient electrolyte additive to stabilize the formation of an SEI layer on mesoporous carbon microbeads (MCMB) in LIBs, especially at elevated temperatures. This electrolyte exhibited an impressive capacity retention of 90.3% after 50 cycles at 0.1 C at elevated temperature, which was remarkably higher than that of the PMA-free electrolyte (83.5%). The improved electrochemical stability at elevated temperatures could be ascribed to the rapid copolymerization of PMA to form a stable and thin SEI layer on the MCMB surface [37].

Herein, we explore the use of butadiene sulfone (BS) as an efficient additive to investigate the electrochemical performance of the electrode/electrolyte interface and SEI formation of the LTO electrode. The chemical structure of BS consists of the double bond and the sulfone functional group (S=O), with a highly electron-withdrawing nature. Our results indicate that BS possesses high conductivity and excellent physical properties. To the best of our knowledge, there is no report on the detailed study of BS as an additive to stabilize the SEI formation for the LTO electrode materials. The effect of the BS additive on the surface morphology and electrochemical performance of the LTO electrode was also described in detail. Moreover, the chemical composition of the SEI film was studied by scanning electron microscopy, attenuated total reflectance infrared spectroscopy (ATR-IR), X-ray photoelectron spectroscopy (XPS), and secondary ion mass spectrometry (SIMS).

## 2. Materials and Methods

A spinel LTO was successfully prepared from the dissolution of lithium acetate dihydrate (CH_3_COOLi∙2H_2_O) (98%, Acros Organics, Geel, Belgium) and tetrabutyl titanate [Ti(OC_4_H_9_)_4_] (99%, Acros Organics, Geel, Belgium) with an Li:Ti molar ratio of 4:5 in ethanol solution (95 wt.%, Shimakyu Co., Ltd., Samut Sakhon, Thailand) under continuous stirring at room temperature for 12 h to obtain the homogeneous solution. The resultant solution was added to the ethanol solution containing citric acid as a chelating agent (99.5%, Acros Organics, Geel, Belgium), and then the resultant mixture solution containing CH_3_COOLi∙2H_2_O: Ti(OC_4_H_9_)_4_: citric acid in the molar ratio of 4:5:1.25 was stirred for another 30 min at room temperature, followed by continuous stirring at 80 °C for 6 h to obtain a gel precursor. The precursor gel was dried at 60 °C for 8 h to remove the residual solvent, subsequently heated at 350 °C for 4 h, and cooled down to room temperature naturally. The collected powder was then calcined at 800 °C for 12 h under an air atmosphere to obtain the LTO. The electrolyte additive solution was prepared by mixing butadiene sulfone (BS; 98%, Acros Organics, Geel, Belgium) with 1 M LiPF_6_ electrolyte in EC/DEC (1:1 vol.) to obtain a 4 wt.% butadiene sulfone solution in an argon-filled glove box. After that 4 wt.% BS solution was diluted to 2 wt.%, 1 wt.%, and 0.5 wt.% butadiene sulfone solution.

The electrode was prepared by mixing the LTO active materials with carbon black and polyvinylidene fluoride (PVDF) (Kynar 7200, Arkema Inc., Khobar, Saudi Arabia) binder in *N*-methyl-2-pyrrollidone (ultra, ISP Technologies Inc., Wayne, NJ, USA) solution. The weight ratio was maintained at 83:10:7. The resultant slurry was coated on Cu foil (100 μm) using the tape casting method and dried under vacuum at 120 °C for 12 h to remove the residual solvent. The mass loading for the LTO electrodes was controlled at ca. 2.2 mg cm^−2^.

Electrochemical tests were performed using CR2032 coin-type cells. The galvanostatic charge/discharge tests were carried out by a battery autotest system (BAT-750B, AcuTech Systems Co., Ltd., Nakuru, Kenya) within a cut-off voltage range of 0.05–2.5 V at room temperature. Electrochemical impedance spectroscopy (EIS) analysis was measured by using the Zahner Zennium electrochemical workstation (Zahner-elektrik GmbH & Co., KG, Kronach, Germany) in the frequency range from 100 kHz to 10 mHz, with an AC amplitude sinusoidal of 5 mV. Before the EIS measurements, the cells were charged/discharged for 3 cycles at a C-rate of 0.2 and an applied voltage of 1.56 V. Before evaluating the structure and composition characterizations, the electrodes were washed three times with ethyl alcohol to remove the residual electrolyte. The surface morphology of the anode before and after cycling performance was analyzed using scanning electron microscopy (SEM) with a JSM-7600 SEM (JEOL Ltd., Tokyo, Japan). The SEI film surface of the LTO electrodes was analyzed by using the ATR-FTIR measurement (Jasco 6700, Jasco Inc., Tokyo, Japan), with wave number ranges between 600 to 4000 cm^−1^. The chemical compositions of the SEI film were confirmed by using X-ray photoelectron spectroscopy (XPS) performed by an ESCALAB 250 spectrometer (Thermo Scientific, Waltham, MA, USA).

## 3. Results and Discussion

**Figure 1:**
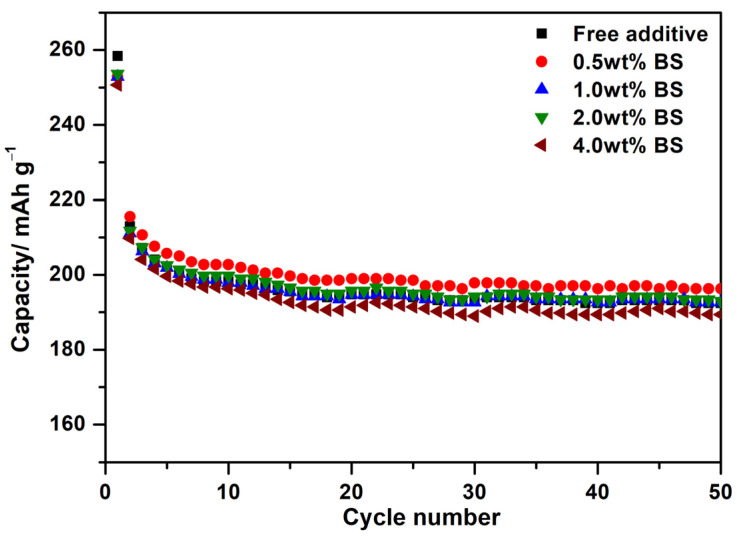
Cycling performances of LTO electrodes with the electrolytes containing different BS concentrations at the 0.2 C-rate.

To investigate the effects of BS additives on the cycling performance of LTO electrodes, a series of electrochemical characterizations were conducted, with the electrolyte containing different BS concentrations. Figure 1 shows the cycling performance of LTO electrodes with the electrolytes containing different BS concentrations at 0.2 C-rate. Using various contents of BS up to 4 wt.%., the initial discharge capacity of the LTO additive-free electrode, and with 0.5 wt.%, 1.0 wt.%, 2.0 wt.%, and 4.0 wt.% BS is found to be 258.3, 253, 252.9, 253.7, and 250.7 mAh g^−1^, respectively. An obvious capacity decay is apparently observed from the first two cycles for all samples, which could be ascribed to the irreversible reaction of SEI film formation. After 50 cycles, the capacity retention of the LTO electrode without the additive, the 0.5 wt.%, 1.0 wt.%, 2.0 wt.%, and 4.0 wt.% BS is reached at 73.3%, 76.4%, 74.9%, 74.8%, and 73.1%, respectively. Among all samples, the LTO electrode in the electrolyte containing 0.5 wt.% BS shows the best stability.

**Figure 2:**
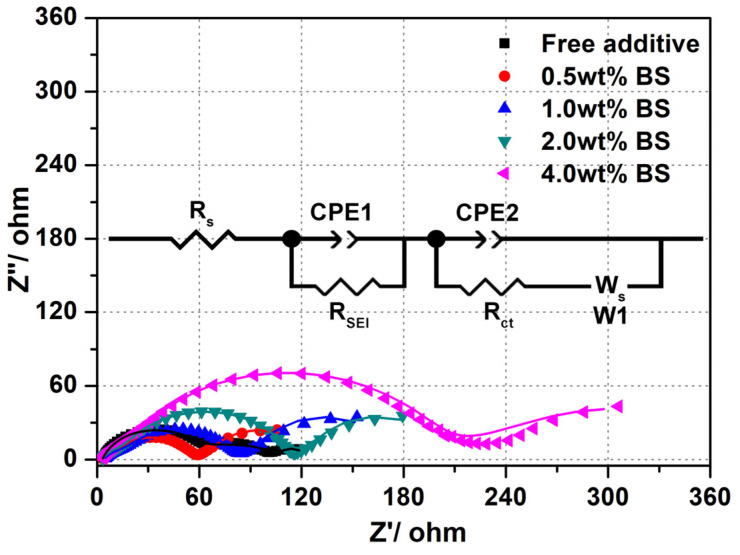
Nyquist plots of the electrodes in the electrolyte with different BS concentrations measured after three consecutive charge/discharge cycles. The inset is the equivalent circuit used to fit the obtained EIS spectra.

To further understand the enhancement of the electrochemical behavior of the SEI film formation and the impedance features of electrodes during cycling, the stabilization and failure mechanisms of 0.5 wt.% BS as an electrolyte additive to the LTO anode was verified by EIS analysis. The Nyquist plots, with different concentrations of BS, are displayed in Figure 2, and the equivalent circuit model illustrated in the inset of Figure 2 is tentatively adopted to fit the EIS spectra. The Nyquist plot consists of two semicircles, at high as well as mid-frequency regions, which correspond to the SEI resistance and Li^+^ migration through SEI (R_SEI_), as well as charge-transfer resistance (R_ct_) between the SEI film and the electrolyte/electrode [28,38], respectively. The R_s_ is related to solution resistance, while the interfacial double layer and passive film capacitance contribute to CPE1 and CPE2, respectively. The slope line at the low-frequency regions is related to the Warburg impedance (W1), which is corroborated by the solid phase diffusion of Li^+^ from the LTO electrode surface. As can be seen form Figure 2, the diameter of the semicircles for LTO in the electrolyte containing 0.5 wt.% BS remarkably decreases when compared to those containing free additives and other BS concentrations due to the SEI film formation. The lower the impedance value, the more rapid the transportation of the Li-ion through the SEI film.

**Figure 3:**
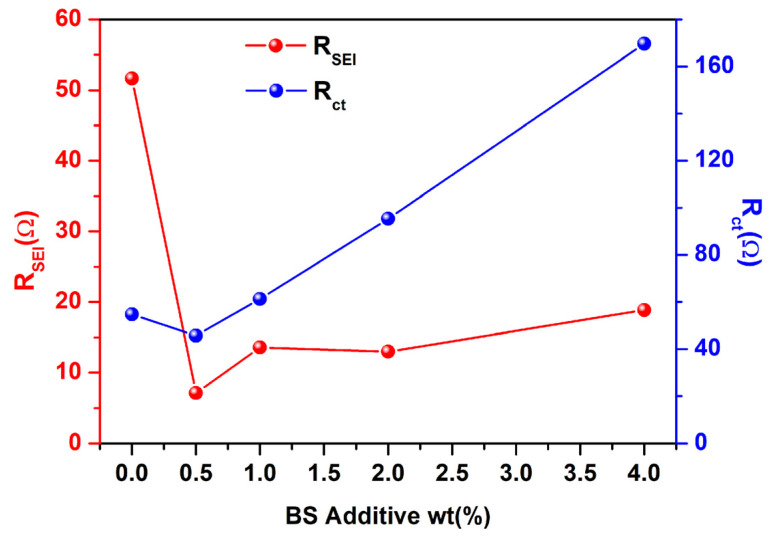
EIS parameters of LTO electrodes, with and without BS additive.

**Figure 4:**
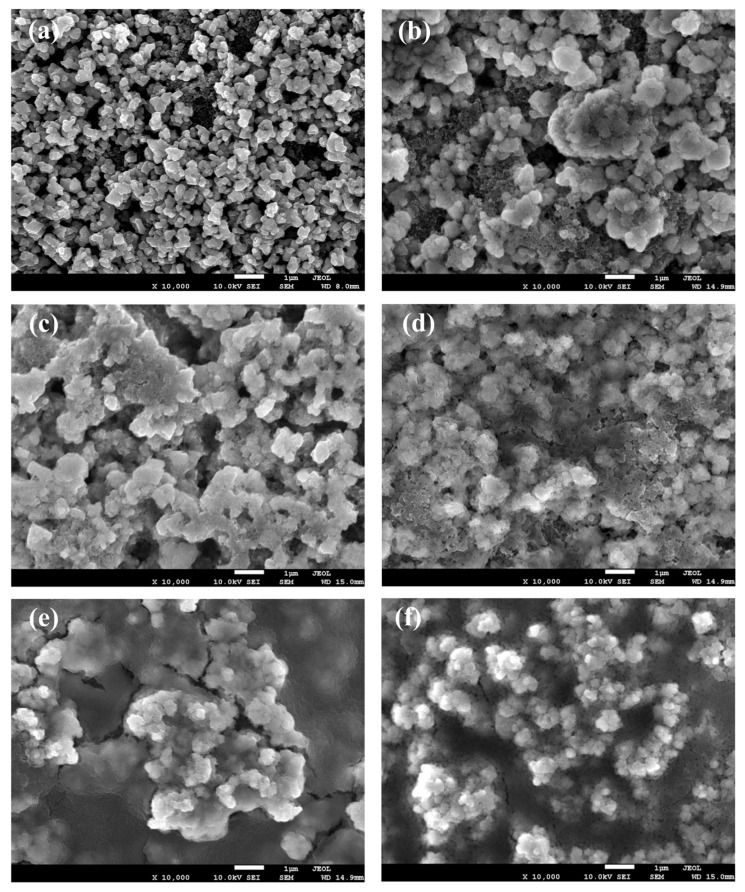
SEM images of LTO electrodes: (**a**) before cycle, (**b**) free additive, (**c**) 0.5, (**d**) 1.0, (**e**) 2.0, and (**f**) 4.0 wt.% BS at the 0.2 C-rate for 50 cycles.

**Figure 5:**
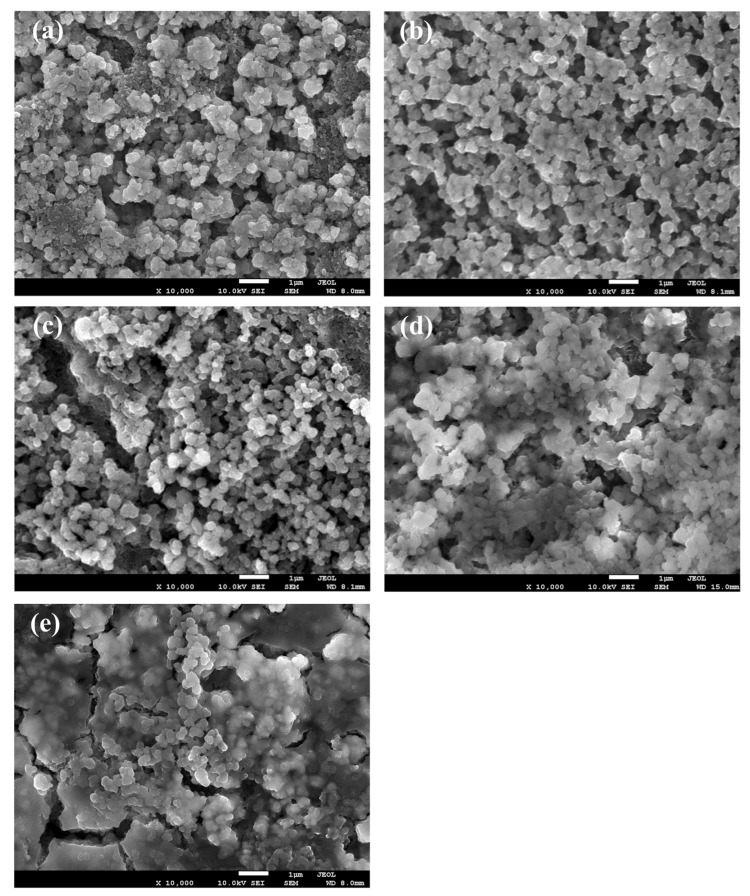
SEM images of (**a**) free additive, (**b**) 0.5 wt.% BS, (**c**) 1.0 wt.% BS, (**d**) 2.0 wt.% BS, and (**e**) 4.0 wt.% BS at the 0.5 C-rate for 50 cycles.

The parameters of the equivalent circuit for different concentrations of BS are recorded in Figure 3. It can be found that the R_s_ values of free additive, the 0.5 wt.%, 1.0 wt.%, 2.0 wt.%, and 4.0 wt.% BS, are 2.89 Ω, 2.86 Ω, 4.30 Ω, 3.08 Ω, and 3.11 Ω after 3 cycles, respectively. The R_SEI_ values of free additive, the 0.5 wt.%, 1.0 wt.%, 2.0 wt.%, and 4.0 wt.% BS are 51.65 Ω, 7.11 Ω, 13.57 Ω, 12.97 Ω, and 18.86 Ω, respectively, indicating that the addition of BS additive in the electrolyte can lower the R_SEI_ values. However, when the concentration of the BS additive is increased, the impedance can drastically increase due to the thick, discontinuous, and cracked surface morphology of the grown SEI film, as shown in Figure 4 and Figure 5. Hence, the chemical composition and the surface morphology of the SEI film play a vital role in impedance [34]. However, adding more BS deeply affects the R_ct_ values (54.73 Ω for additive free; 45.71 Ω for 0.5 wt.%; 61.31 Ω for 1.0 wt.%; 95.40 Ω for 2 wt.%; 169.70 Ω for 4 wt.%). This indicates that the lower impedance value is related to the rapid movement of Li^+^ through the SEI film. Therefore, the results indicate that BS has the benefit of an SEI film. However, increasing the BS amount leads to the retardation of the charge transfer reaction at the interface of the electrolyte and the electrode system. According to Aurbach et al. [39], the generation of Li_2_CO_3_ and Li_2_SO_3_ can effectively create the passive film over the LTO electrode surface, thus resulting in the reduction of the interfacial impedance of the SEI film, which can increase the electrochemical performances.

Figure 4 shows the surface morphology of SEI film grown on LTO electrodes in the presence of LiPF_6_ and EC/DEC at different concentrations of BS at the 0.2 C-rate. The surface morphology, before and after the cycles, is significantly different, as shown in the SEM images. From the SEM analysis, the natural LTO surface is composed of the spherically shaped particles, as seen in Figure 4a. The surface morphology of the fresh electrode is reasonably different from the additive-free electrode, as shown in Figure 4a,b. Compared to the fresh electrode, the surface of the additive-free electrode is very coarse, with irregular particles. It can be confirmed that the difference in the amount of BS additive affects the surface morphology and the SEI film quality. Notably, the surface of the LTO electrode with the addition of 0.5 wt.% of BS cycled at the 0.2 C-rate shows a compact and rigid surface. Figure 4b and c shows the similar surface morphology to the pristine LTO electrode, which indicates that the addition of 0.5 wt.% BS effectively controls the formation of SEI film during the cycling performance, therefore leading to enhanced electrochemical performance. Moreover, there are few fractures found in the electrodes with a high amount of BS additive. SEI film growth is significantly increased by increasing the amount of BS beyond 1.0 wt.%, as it can be seen that the LTO surface was entirely covered by a thick layer of SEI film. When increasing the BS content in the electrolyte system, the SEI film covers the entirely electrode surface with the cracked surface morphology on the LTO electrodes, as shown in Figure 4c–f. Moreover, Figure 5a–e displays the LTO electrodes with different concentrations of BS, which were charged/discharged at the 0.5 C-rate. At the 0.5 C-rate, we can find that the electrodes with the addition of 2.0 wt.% and 4.0 wt.% BS possess a distinct morphology from the other electrodes because BS can promote SEI film formation on the LTO electrode. Subsequently, the film quality is lost after cycling. From the obtained results, we suggest that the smooth and stable SEI film can help the Li-ion insertion/extraction to/from the LTO electrode. However, when the SEI film is too thick, the charge transfer resistance increases [31]. Therefore, the results of EIS indicate that the BS electrolyte additive can improve the SEI film, but the charge transfer cannot occur effectively when the thick SEI film is formed.

**Figure 6:**
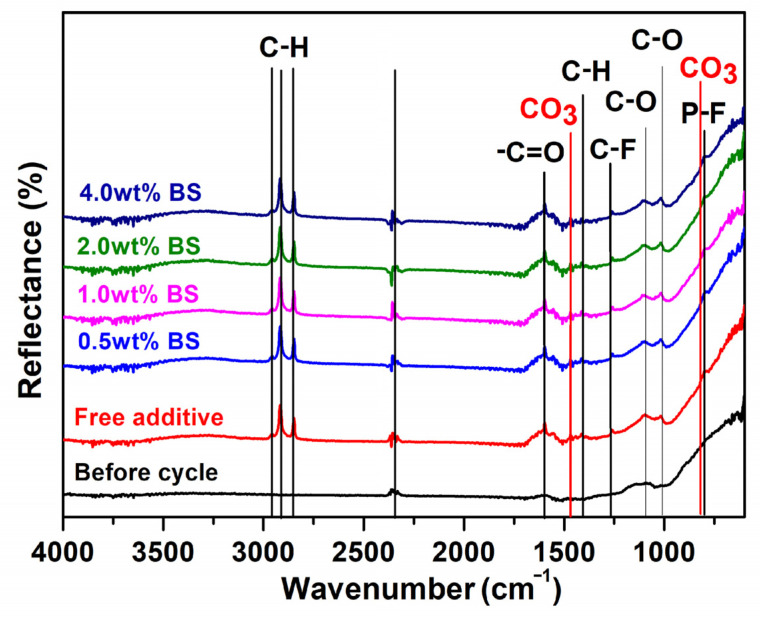
ATR-FTIR spectra of LTO, before and after cycling, in different concentrations of BS contents at the 0.2 C-rate for 50 cycles.

In order to understand the information regarding the SEI film in detail when adding BS electrolyte additive in the LTO electrode, the surface composition analysis was carried out by ATR-FTIR and XPS after cycling in the electrolyte, with and without BS. Figure 6 displays the ATR-FTIR measurement of the LTO electrodes with the different concentrations of BS additive. From the results, some organic groups, before and after cycling, can be found in all the electrodes. For the before-cycled electrode, the organic groups might be carbon black and binder (polyvinylidene fluoride). After 50 charges/discharge cycling process, the new signal appears, which is attributed to electrolyte decomposition resulting in the formation of the SEI film. The results are presented in Table S1. The results indicate the formation of different organic groups. The peak located at 798 cm^−1^ (P-F) is attributed to Li_x_PF_y_ originating from the LiPF_6_ decomposition [40]. The peaks at 1039 cm^−1^ (C–O), 1600 cm^−1^ (–C=O), 1405 cm^−1^ (O–CO_2_), and 2800–2950 cm^−1^ (C–H) are the characteristic peaks of ROCO_2_Li. The peaks at 1476 and 836 cm^−1^ (CO_3_) are attributed to the asymmetric stretch vibration and out-of-plane bend vibration of Li_2_CO_3_, respectively [41]. The peaks around 1405 cm^−1^ (C–H overlapped with O–CO_2_) and 1310 cm^−1^ (C–F) might develop from the PVDF binder. From the results, the decomposed EC and DEC, combined with Li^+^, produce the organic species such as ROCO_2_Li and Li_2_CO_3_ [16]. The resolution of the ATR-FTIR analysis range is 5 μm [39], but the SEI thickness can form only in nanometer scale. Therefore, the results obtained from the additive-containing electrodes are similar to those obtained from the free additive.

**Figure 7:**
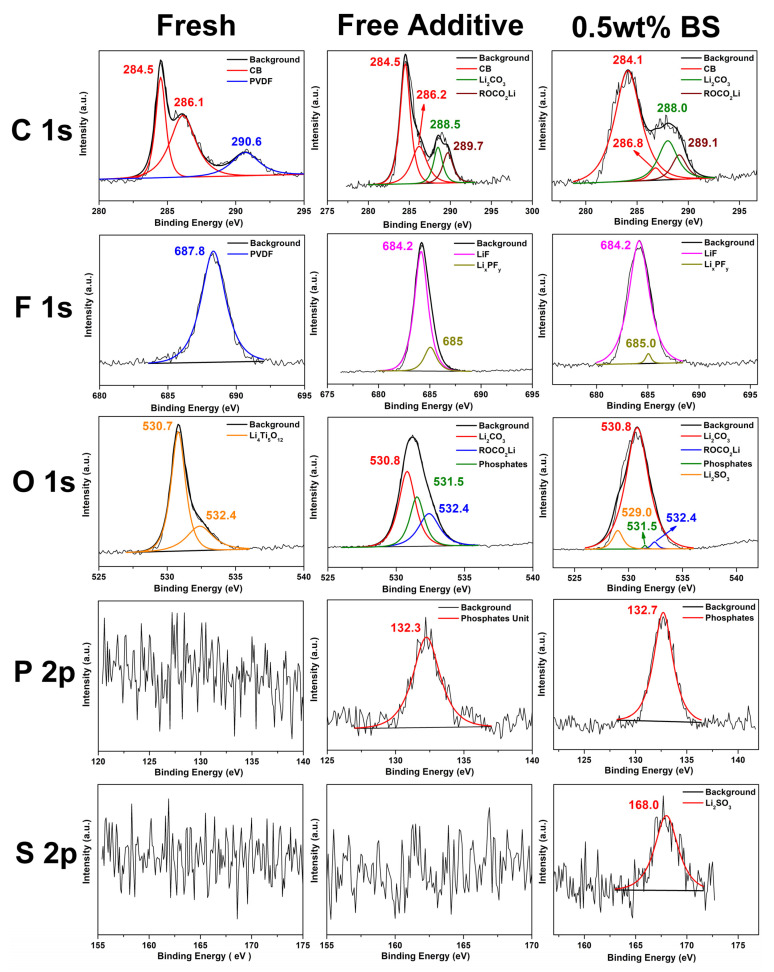
C 1s, F 1s, O 1s, P 2p, and S 2p XPS spectra of the fresh LTO electrode and the cycled electrode, without and with 0.5 wt% BS additive at 0.2 C for 50 cycles.

To extensively understand the SEI chemical composition of the LTO electrode surface during the cycling performance analysis, ex situ XPS studies were employed to analyze the fresh electrode and electrodes cycled in the electrolyte containing free additive, and 0.5 wt.% BS additive, as depicted in the Figure 7. The binding energies of the chemical products identified in the various XPS analyses are listed in Table S2. It can be seen from Figure 7 that the compositions of the cycled electrodes are different from that of the fresh electrode, showing that the SEI has been formed on LTO. From the fresh and free additive results, the C 1s spectra of the fresh electrode show binding energy at 284.5 and 286.1 eV corresponding to the sp^2^ and sp^3^ carbon of carbon black (CB), respectively, whereas the peak at 290.6 eV is assigned to the –CF_2_ group in PVDF. In the electrodes after cycling, the C 1s spectrum shows new carbon species forming on the LTO electrode surface. The peaks at 289.7 and 291.6 eV correspond to the formation of Li_2_CO_3_ and ROCO_2_Li compounds, respectively, that are originated from EC and DEC decomposition during the charge/discharge process [42]. As previously reported, Li_2_CO_3_ is the most important species of the organic-based electrolytes. The peak intensity of Li_2_CO_3_ is very low for LTO electrodes containing BS additive, which clearly illustrates that BS efficiently suppresses the decomposition of the carbonate-based electrolytes forming the fragile SEI film compared to the additive free and fresh LTO electrodes. These results are in good agreement with the EIS measurement, showing a lower Rct value of the LTO electrode with the addition of 0.5 wt.% BS additive.

From the F 1s spectra, the peak at 687.8 eV comes from the PVDF binder, while the peaks at 684.2 and 685.0 eV are assigned to LiF and Li_x_PF_y_, respectively [43]. The O 1s spectra show the binding energy of 530.7 and 532.4 eV corresponding to LTO electrode. After cycling, the electrodes show the formation components with the binding energy of 529.0, 530.8, 531.5, and 532.4 eV corresponding to Li_2_SO_3_, Li_2_CO_3_, phosphates, and lithium alkyl carbonates, respectively [44]. The P 2p spectra, with the binding energy of around 132.5 eV, suggest the phosphate groups on the LTO electrode. In the S 2p spectra, the peak at 168.0 eV is assigned to Li_2_SO_3_ [44,45]. After cycling, the subsequent electrode reactions may take place in the electrolyte and the additive medium, as shown below.

2DEC + 2Li + 2e^−^ → RCO_2_Li + ROCO_2_Li + C_4_H_10 (g)_ (1)

2EC + 2Li + 2e^−^ → RCO_2_Li + ROCO_2_Li + Li_2_CO_3_ + C_2_H_4 (g)_ (2)

LiPF_6_ + H_2_O → LiF+ PF_5_ + H_2_O (3)

PF_5_ + Li_2_CO_3_ → 2LiF + POF_3_ + CO_2 (g)_ (4)

LiPF_6_ + H_2_O → LiF + POF_3_ + 2HF (5)

POF_3_ + ne^−^ + nLi^+^ → LiF + Li_x_POF_y_ (6)

BS + Li + e^−^ → Li_2_SO_3_ + C_4_H_8 (g)_ (7)

For the fresh and after cycled electrode, the new components at the C 1s, F 1s, O 1s, and P 2p spectra were detected, suggesting the SEI formation on the LTO electrode due to EC, DEC, and LiPF_6_ decomposition when discharged to 0.05 V. Sulfur-containing species were detected on the electrode with the addition of 0.5 wt.% BS electrolyte additive, further confirming the reduction of the additives incorporated into the SEI. Therefore, the composition of SEI is changed. The sulfur-containing additive BS makes SEI film more stable and smoother, as shown in Figure 4 and Figure 5. Furthermore, 0.5 wt.% BS additive can decrease the electro-resistance of the SEI film and improve the cycling performance of the LTO/Li cells at room temperature. It is significant to remember that the chemical composition varies depending upon the change in the surface morphology on the modified electrode surface, as shown in Figure 4 and Figure 5.

## 4. Conclusions

To summarize, this study helps provide new insights into the SEI formation on the LTO anode in a lithium-ion battery, with EC/DEC based electrolyte-containing BS as a novel additive. It was observed that the SEI film consists of inorganic sulfite and alkyl sulfite species. By adding a 0.5 wt.% BS additive into the electrolyte, the generation of the organic and inorganic species, such as LiF, Li_2_CO_3_, ROCO_2_Li, Li_2_SO_3_, and ROSO_2_Li, was prevented, and a fragile SEI film was formed over the LTO surface. The sulfur-containing electrolyte can provide Li_2_SO_3_ in the SEI film, acting as an accelerator to facilitate the stable and smooth SEI film formation, building a more protective SEI film. In the presence of 0.5 wt.% BS, the SEI electro-resistance decreased from 51.65 to 7.11 Ω when LTO was discharged to 0.05 V, and the discharge capacity increased to 3.1% at a voltage window of 0.05–2.5 V. In contrast, when adding 4 wt.% BS in LTO/Li half cells, the electro-resistance of the charge transfer increased to 169.70 Ω due to the thick SEI film. Our work suggests that BS is a possible additive for future LIB applications, efficiently stabilizing SEI formation on the LTO electrode when operated within the large voltage window of 0.05–2.5 V.

## Data Availability

Not applicable.

## Supplementary Materials

The following supporting information can be downloaded at: https://www.mdpi.com/article/10.3390/polym15081965/s1, Table S1: The assignments of the characteristic bands observed in ATR-FTIR spectra; Table S2: The assignments of the characteristic bands observed in XPS spectra.
