# Supplementary material for: Effects of Butadiene Sulfone as an Electrolyte Additive on the Formation of Solid Electrolyte Interphase in Lithium-Ion Batteries Based on Li4Ti5O12 Anode Materials
Source: Polymers (Basel). 2023 Apr 21;15(8):1965. doi: 10.3390/polym15081965 (PMC10143351; doi:10.3390/polym15081965)
Supplement: Supplementary file 1 [file polymers-15-01965-s001.zip › polymers-2268435-supplementary.pdf]

Supporting Information

Effects of Butadiene Sulfone on the Formation of Solid Electrolyte Interphase in Lithium-ion Batteries based on $Li_4Ti_5O_{12}$ Anode Materials

Yu-Ruei Kung*[,1], Cheng-Yao Li[1], Panitat Hasin*[,2] Chia-Hung Su [3] and Jeng-Yu Lin [3,4,*]

*Corresponding authors
[1] Department of Chemical Engineering and Biotechnology, Tatung University (TTU), 40. Sec.3, Zhong Shan N. Rd., Taipei, 104327, Taiwan (R.O.C.)
[2] Department of Chemistry and Center of Excellence for Innovation in Chemistry (PERCH-CIC), Ministry of Higher Education, Science, Research and Innovation, Faculty of Science, Kasetsart University, Bangkok 10900, Thailand
[3] Research Center for Chinese Herbal Medicine, Ming Chi University of Technology, New Taipei City 24301, Taiwan
[4] Department of Chemical and Materials Engineering, Tunghai University (THU), No. 1727, Sec. 4, Taiwan Boulevard, Xitun District, Taichung City 407224, Taiwan (R.O.C)
E-mail: yrkung@gm.ttu.edu.tw; fscipths@ku.ac.th; jylin@thu.edu.tw

Table S1 The assignments of the characteristic bands observed in ATR-FTIR spectra

Band-description	Transmittance Peak (cm^{-1})	Possible assignments
P−F	798	Li_xPF_y
C−H	2800-2950	$ROCO_2Li$
C−O	1039	$ROCO_2Li$
−C=O	1600	$ROCO_2Li$
C−F	1310	PVDF
C−H, O−CO_2 (overlapped)	1405	$ROCO_2Li$
CO_3 (asymm. str.)	1476	Li_2CO_3
CO_3 (out-of plane vib.)	836	Li_2CO_3
CO_2	1476	$ROCO_2Li$

Table S2 The assignments of the characteristic bands observed in XPS spectra

Peak Position	Binding energy BE (eV)	Assignment	Reference
**C 1s**	284.5	sp^2 carbon of carbon black (CB)	16, 39, 42
	286.1	sp^3 carbon of carbon black (CB)	
	290.6	PVDF	
	289.7	Li_2CO_3	
	291.6	$ROCO_2Li$	
**F 1s**	687.8	PVDF	42, 43
	684.2	LiF	
	685.0	Li_xPF_y	
**O 1s**	530.7	LTO	42, 44
	532.4	LTO	
	529.0	$LiSO_3$	
	530.8	Li_2CO_3	
	531.5	Phosphate group	
	532.4	$ROCO_2Li$	
**P 2p**	132.5	Phosphate group	44
**S 2p**	168.0	Li_2SO_3	44,45
